# Molecular characterization and immune modulation properties of *Clonorchis sinensis*-derived RNASET2

**DOI:** 10.1186/1756-3305-6-360

**Published:** 2013-12-23

**Authors:** Yanquan Xu, Wenjun Chen, Meng Bian, Xiaoyun Wang, Jiufeng Sun, Hengchang Sun, Feifei Jia, Chi Liang, Xuerong Li, Xiaonong Zhou, Yan Huang, Xinbing Yu

**Affiliations:** 1 Department of Parasitology, Zhongshan School of Medicine, Sun Yat-sen University, Guangzhou 510080, People’s Republic of China; 2 Key Laboratory for Tropical Diseases Control, Ministry of Education, Sun Yat-sen University, Guangzhou 510080, People’s Republic of China; 3 National Institute of Parasitic Diseases, Chinese Center for Disease Control and Prevention, Shanghai 200025, People’s Republic of China

**Keywords:** *Clonorchis sinensis*, RNASET2, T2 ribonuclease, Excretory/secretory product, Dendritic cell, Immune modulation

## Abstract

**Background:**

*Clonorchis sinensis* (*C. sinensis, Cs*) is a trematode parasite that often causes chronic cumulative infections in the hepatobiliary ducts of the host and can lead to pathological changes by continuously released excretory/secretory proteins (ESPs). A T2 ribonuclease in trematode ESPs, has been identified as a potent regulator of dendritic cell (DCs) modulation. We wondered whether there was a counterpart present in *Cs*ESPs with similar activity. To gain a better understanding of *Cs*ESPs associated immune responses, we identified and characterized RNASET2 of *C. sinensis* (*Cs*RNASET2) in this paper.

**Methods:**

We expressed *Cs*RNASET2 in *Pichia pastoris* and identified its molecular characteristics using bioinformatic analysis and experimental approaches. The immune modulation activities of *Cs*RNASET2 were confirmed by evaluating cytokine production and surface markers of recombinant *Cs*RNASET2 (r*Cs*RNASET2) co-cultured DCs, and monitoring levels of IgG isotypes from r*Cs*RNASET2 administered BALB/c mice.

**Results:**

*Cs*RNASET2 appeared to be a glycoprotein of T2 ribonuclease family harboring conserved CAS motifs and rich in B-cell epitopes*.* Furthermore, *Cs*RNASET2 was present in *Cs*ESPs and was able to modulate cytokine production of DCs. In addition, r*Cs*RNASET2 could significantly suppress the expression of lipopolysaccharide-induced DCs maturation markers. In addition, when subcutaneously administered with r*Cs*RNASET2 there was a marked effect on IgG isotypes in mouse sera.

**Conclusion:**

Collectively, we revealed that *Cs*RNASET2, a T2 ribonuclease present in *Cs*ESPs, could modulate DCs maturation and might play an important role in *C. sinensis* associated immune regulation in the host.

## Background

Clonorchiasis, caused by the infection of *Clonorchis sinensis* (*C. sinensis*), is highly epidemic in several Asian countries. More than 35 million people are infected with *C. sinensis* globally [1]. Clonorchiasis is predominantly caused by ingesting raw or undercooked freshwater fish harboring metacercariae [2]. When ingested by the host, larvae excyst in the duodenum and then migrate into the peripheral intrahepatic bile ducts to develop as adult worms [3]. *C. sinensis* adult worms can thrive for more than 10 years in humans [4]. People infected with *C. sinensis* are often asymptomatic, however, repeated and chronic infections can eventually lead to various hepatobiliary symptoms and complications. During the long term of parasitism, the liver flukes continuously release excretory/secretory proteins (ESPs), a cocktail of hundreds to thousands of bioactive proteins. Prior studies have demonstrated that the components of ESPs from *C. sinensis* (*Cs*ESPs) are implicated in biological processes especially the induction of host immune response [5], which may be intimately associated with formation of cholangitis, liver cirrhosis and cholangiocarcinoma (CCA) [6]. Owing to its carcinogenicity, *C. sinensis* has been regarded as a group I carcinogen of cholangiocarcinoma [7].

T2 ribonucleases are transferase-type RNase and distribute broadly in almost all groups of living organisms including bacteria, fungi, virus, plants, and animals [8]. All of these enzymes have diverse biological activities, such as degradation of self-RNA, clearance of nucleic acids, serving as extra- or intracellular cytotoxins, and regulation of host immune responses [8-10]. Numerous studies have been conducted to characterize the biochemical properties and immunoregulatory roles of a T2 ribonuclease (omega-1) in *Schistosoma mansoni* (*Sm*omega-1), which is present in soluble egg antigens (SEA) of *S. mansoni*, formed by egg released ESPs [11-13]. *Sm*omega-1 could trigger Th2 response by modulating dendritic cells (DCs) phenotype, structure and protein synthesis [14-16]. Moreover, *Sm*omega-1 has been reported to induce priming of Foxp3^+^ Tregs, which play crucial immunoregulatory roles in the process of *S. mansoni* infection [17]. Interestingly, the function of *Sm*omega-1 to enhance Foxp3 expression also depends on the alteration of DCs. These findings illustrate that *Sm*omega-1 is a key component of ESPs to modulate host immune responses, which is elicited by DCs alteration. DCs are defined as professional antigen-presenting cells serving as the sentinels of the immune system and have the unique capacity to induce and coordinate both innate and adaptive immune responses [18-22]. These findings give us insight into interactions between a secreted antigen and DCs, leading us to gain a better understanding of helminth associated immune responses.

In the present study, we expressed and characterized *C. sinensis* RNASET2 (*Cs*RNASET2). We investigated the effects of r*Cs*RNASET2 on bone marrow derived dendritic cell (BMDCs) modulation. In addition, we evaluated humoral immune responses initiated by r*Cs*RNASET2 in BALB/c mice. Based on these observations, we proposed that *Cs*RNASET2 was intimately associated with *C. sinensis*-triggered immune response in the host.

## Methods

### Animals

Female 6- to 8-week-old BALB/c mice and SD rats were obtained from the animal center of Sun Yat-Sen University (Guangzhou, China). The animals were housed in a pathogen-free facility. All animal experiments were conducted under Animal Care and Use Committee of Sun Yat-sen University (Permit No: SCXK (Guangdong) 2009–0011).

### Sequence analysis of *Cs*RNASET2

The full-length encoding sequence of *Cs*RNASET2 [Accession No. GAA50115.1] was downloaded from GenBank in NCBI [http://www.ncbi.nlm.nih.gov/]. Homology analysis was performed using the blastx program [http://blast.ncbi.nlm.nih.gov/]. The molecular characteristics and the functional domains of *Cs*RNASET2 were assessed using proteomics tools provided by Expasy website [http://www.expasy.org/]. The alignments of the deduced amino acid sequence and T2 amino acid sequences from other species were performed by the software Vector NTI suite 8.0.

### Preparation of antigens and polyclonal antibodies

Recombinant *Cs*RNASET2 was expressed in *P. pastoris* according to the manufacturer’s protocol (Invitrogen, USA). RNase activity of r*Cs*RNASET2 was identified, and deglycosylation assay of the protein was carried out with PNGase F (New England BioLabs, USA). We collected *Cs*ESPs referring to the protocol as previously described [23]. Each BALB/c mouse was immunized subcutaneously with 100 μg r*Cs*RNASET2 or ESPs, which were respectively emulsified with complete Freund’s adjuvant at the first injection. Two booster injections at 2-week intervals were performed with 50 μg of proteins, which were emulsified with incomplete Freund’s adjuvant. After 2 weeks of the final boosting, anti-sera were collected and antibody titers were determined by ELISA. All anti-sera samples were split and stored at - 80°C.

### Western blotting

r*Cs*RNASET2 and *Cs*ESPs were subjected to 12% SDS-PAGE, and subsequently electrotransferred onto the PVDF membranes (Millipore, USA) for 1 h at 100 V. The membranes were then blocked with 5% skimmed milk in PBS for 2 h at room temperature, and incubated with myc-epitope monoclonal antibody (1:1000 dilutions), his-tag monoclonal antibody, mouse anti-r*Cs*RNASET2 sera (1:2000 dilutions), mouse anti- ESPs sera, naïve mouse and *C. sinensis*-infected mouse sera (1:100 dilutions) respectively. The membranes were then incubated with peroxidase-conjugated goat anti-mouse IgG (Proteintech, USA, 1:10,000 dilutions) for 1 h at room temprature. The reactions were visualized by enhanced chemiluminescence (ECL) method.

### Generation and stimulation of bone marrow derived dendritic cells

Femurs were obtained from BALB/c mice and the muscle tissues were removed with scissors. The bone marrow cells (BMCs) were isolated by flushing the bone marrow with chilled RPMI-1640 medium. Red blood cells were lysed with red blood cell lysing buffer (Sigma, USA). The remaining BMCs were washed twice and then resuspended into 2 × 10^6^/ml. BMCs were cultured in complete RPMI-1640 supplemented with 20 ng/ml mouse GM-CSF (R&D Systems, USA) and 10 ng/ml mouse IL-4 (R&D Systems). On days 3 and 5, fresh culture medium including the supplements were added. Immature BMDCs were harvested on day 7 and stimulated with or without different concentrations of r*Cs*RNASET2 (0.05-50 μg/ml) or ESPs (10–160 μg/ml) in the presence of 1 μg/ml LPS (sigma) for 12, 24 and 48 h. Generally, Th1- and Th2-polarizing Ag stimulate DCs via discrete pathways, coupled with distinct modifications of the DC maturation. Therefore, we utilized r*Cs*FABP, a protein has been reported to induce Th1 immune responses, as a control protein [24,25].

### Flow cytometry

Surface markers on pretreated BMDCs were detected by FCM using the following mAbs: FITC labeled anti-CD11c (eBioscience, USA), PE labeled anti-CD80, anti-CD86, APC labeled anti-CD40 (BD PharMingen, USA). For staining, BMDCs were washed twice with PBS containing 0.1% BSA and 0.05% sodium azide. The cells were thereafter incubated with the respective mAbs for 30 min at 4°C in the dark. Then, cells were washed twice and resuspended in PBS. Flow cytometry was performed on a Beckman Coulter Gallios cytometer and analyzed using the Kaluza software (Beckman Coulter, USA).

### ELISA

Cell-free supernatants were harvested at different time points after stimulation, and levels of IL-12p70 and IL-10 were assessed by commercially available ELISA kits (eBioscience) according to the manufacturer’s protocol. Sera were collected from r*Cs*RNASET2 immunized mice or PBS immunized mice at 4 and 6 weeks after primary injection. Levels of IgG1 and IgG2a in sera were evaluated by ELISA coated with 100 μl 5 μg/ml r*Cs*RNASET2 each well overnight at 4°C. ELISA plates were blocked with 5% skimmed milk for 2 h at 37°C and then incubated with sera for 2 h at 37°C. Subsequently, the plates were incubated with HRP-conjugated goat anti-mouse IgG1 and IgG2a (invitrogen) for 1 h at 37°C. The optical density was tested at 450 nm.

### Statistical analysis

Data were routinely analyzed by the GraphPad Prism 5 software. The Mann–Whitney test was performed to calculate statistical significance of differences between two groups of observations. *p* -Values < 0.05 were considered statistically significant.

## Results

### Sequence analysis of *Cs*RNASET2

**Figure 1:**
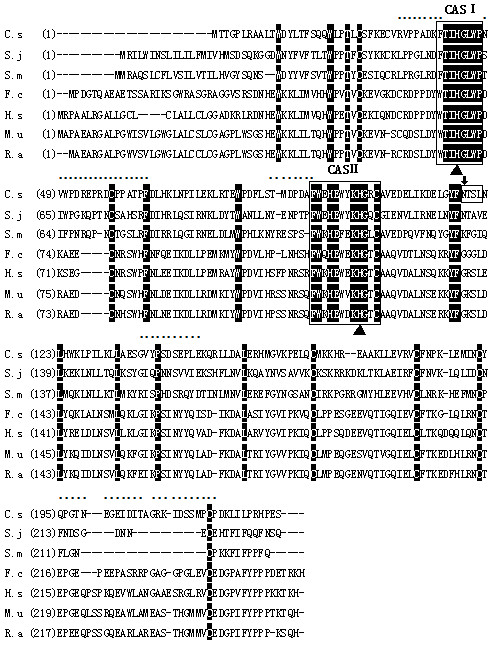
**Sequence alignment of RNASET2 from *C. sinensis *with homologues from other species.** The identical amino acids of all aligned sequences are shaded in black. The conserved ribonuclease T2 CAS I and CAS II domains are marked with box1 and box2. Two highly conserved histidine residues in the CAS regions are indicated by black triangles. A N-linked glycosylation site is marked with box3 and an arrow. The B cell epitopes are labeled by dots. GenBank Accession Numbers: C.s, *Clonorchis sinensis*, [GAA50115.1]; S.j, *Schistosoma japonicum*, [AAW26577.1]; S.m, *Schistosoma mansoni*, [ABB73003.1]; F.c, *Felis catus*, [XP_003986767.1]; H.s, *Homo sapiens*, [AAH51912.1]; M.u, *mus musculus*, [AAI00331.1]; R.a, *rattus norvegicus*, [NP_001099680.1].

The complete coding sequence of *Cs*RNASET2 contained 690 bp encoding a putative protein of 229 amino acids with the predicted molecular weight of 26.6 kDa. The hypothetical pI of the deduced protein was 5.65. The estimated half-life of *Cs*RNASET2 was more than 20 hours in yeast and more than 10 hours in *E. coli*. The instability index of the deduced protein was 35.40, indicating *Cs*RNASET2 might be a stable protein. Blastx analysis showed that the sequence was a glycoprotein of the T2 ribonuclease family, which contained two critical histidine residues in the CAS motifs. However, the sequence just shared 43% and 39% identity with its homologues from *S. janpani* and *S. mansoni*, and it was rich in B-cell epitopes, which might suggest *Cs*RNASET2 as being a good immunogen (Figure 1).

### Identification of *Cs*RNASET2 as a component of *C. Sinensis* ESPs

**Figure 2:**
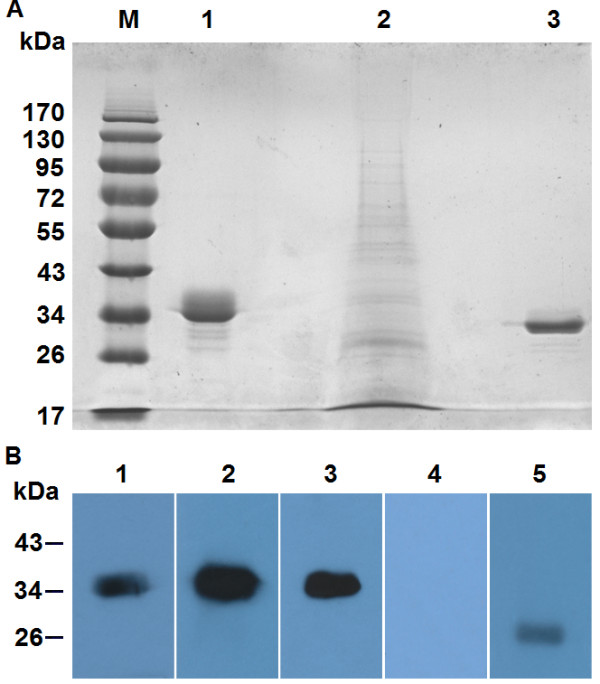
**Determination of *Cs*RNASET2 as a component of *C. Sinensis *ESPs. (A)** The deglycosylation assay of r*Cs*RNASET2 was evaluated by SDS-PAGE protein molecular weight markers (M); untreated r*Cs*RNASET2 (lane 1); *Cs*ESPs (lane 2); r*Cs*RNASET2 incubated with PNGase F deglycosylation enzyme (lane 3). **(B)** Western blotting analysis of r*Cs*RNASET2. The r*Cs*RNASET2 reacted with sera from mice infected with *C. sinensis* (lane 1); mice immunized with r*Cs*RNASET2 (lane 2), mice immunized with *Cs*ESPs (lane 3) and naïve mouse (lane 4); *Cs*ESPs reacted with sera from mice immunized with r*Cs*RNASET2 (lane 5). One of four independent experiments is shown.

We successfully expressed r*Cs*RNASET2 (Additional file 1: Figure S1A) and identified its c-myc epitope and his-tag by western blot (Additional file 1: Figure S1B). The RNase activity of r*Cs*RNASET2 was determined (Additional file 1: Figure S2). We noted that deglycosylated r*Cs*RNASET2 displayed ~4 kDa reduction of molecular weight (Figure 2A). In addition, r*Cs*RNASET2 could be probed by anti-*Cs*RNASET2, anti-*Cs*ESPs and *C. sinensis*-infected mouse sera at a prominent single band around 35 kDa, whereas the protein could not be recognized by sera from naïve mice. In addition, *Cs*ESPs reacted with mouse anti-*Cs*RNASET2 sera at a single band around 27 kDa (Figure 2B). These results indicated that r*Cs*RNASET2 was indeed a component of *Cs*ESPs with glycosylation and RNase activity.

### Cytokine production of BMDCs in response to r*Cs*RNASET2

**Figure 3:**
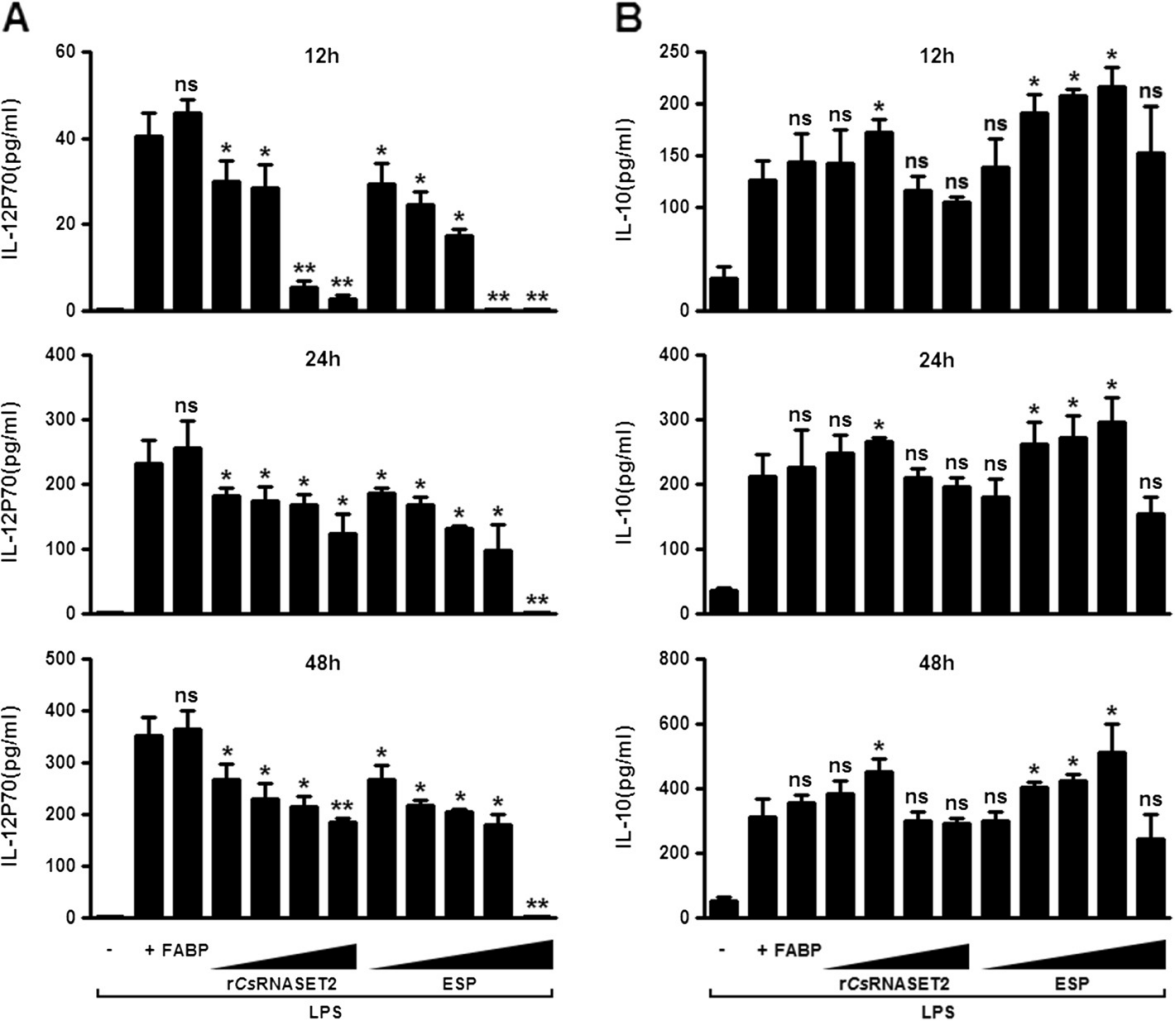
**Cytokine production by BMDCs in response to r*Cs*RNASET2.** BMDCs (2 × 10^6^/ml) were incubated with 1 μg/ml LPS in the presence or absence of various concentrations of (10, 20, 40, 80 and 160 μg/ml) ESPs, (0.05, 0.5, 5 and 50 μg/ml) r*Cs*RNASET2 or (0.5 μg/ml) r*Cs*FABP. Supernatants were harvested at different time points after stimulation and evaluated by ELISA for expression of IL-12p70 **(A)** and IL-10 **(B)**. Error bars represent mean cytokine concentrations ± SD (n = 5). Statistical significance was analyzed by the Mann–Whitney test (*p < 0.05, **p < 0.01, ns: not significant).

IL-12p70 and IL-10 are potent adjustive cytokines in the immune system, which can be secreted by DCs [26,27]. We incubated BMDCs with LPS in the presence or absence of various concentrations of r*Cs*RNASET2, ESP or 0.5 μg/ml r*Cs*FABP. We found that both r*Cs*RNASET2 and *Cs*ESPs suppressed the LPS-induced up-regulation of IL-12p70 on BMDCs in a dose-dependent manner at the indicated time points (Figure 3A). In contrast, *Cs*ESPs (20–80 μg/ml) enhanced the expression of IL-10 in a dose-dependent manner, and r*Cs*RNASET2 could promote IL-10 synthesis only at a concentration of 0.5 μg/ml (Figure 3B). In addition, r*Cs*FABP could not obviously interfere with the cytokine production of DCs.

### Effects of r*Cs*RNASET2 on BMDCs phenotypic modifications

**Figure 4:**
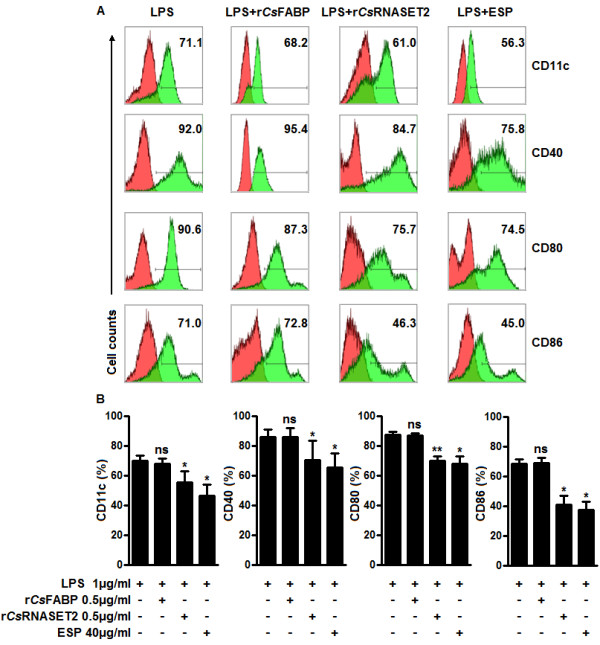
**Modulation of BMDCs maturation elicited by r*Cs*RNASET2. (A)** Days-7 BMDCs (2 × 10^6^/ml) were stimulated with or without 0.5 μg/ml r*Cs*RNASET2, 0.5 μg/ml r*Cs*FABP or 40 μg/ml ESPs in the presence of 1 μg/ml LPS for 24 h. The levels of CD11c, CD40, CD80 and CD86 on BMDCs were detected by flow cytometry. Values stand for the percentage of treated cells staining positive for the indicated marker. Histograms are representative of seven independent experiments. **(B)** Statistical results of the expression of surface markers on BMDCs. Data are expressed as mean ± SD. Statistical significance was analyzed by the Mann–Whitney test (*p < 0.05, **p < 0.01, ns: not significant).

A previous study has demonstrated that helminth antigens can affect the maturation of DCs [28]. To confirm the effects of *Cs*RNASET2 on DCs maturation, BMDCs were treated with LPS in the presence or absence of r*Cs*RNASET2, r*Cs*FABP (control protein) or ESPs for 24 h, and a series of surface markers expressed on DCs were assessed. In the presence of LPS alone, the expression levels of CD11c, CD40, CD80 and CD86 on BMDCs were raised, when r*Cs*RNASET2 or ESPs were added, the LPS-triggered up-regulation of surface markers was strikingly reduced. In contrast, r*Cs*FABP had no significant effect on the expression of these surface markers (Figure 4A, B).

### IgG isotype analysis of r*Cs*RNASET2 immunized mice

**Figure 5:**
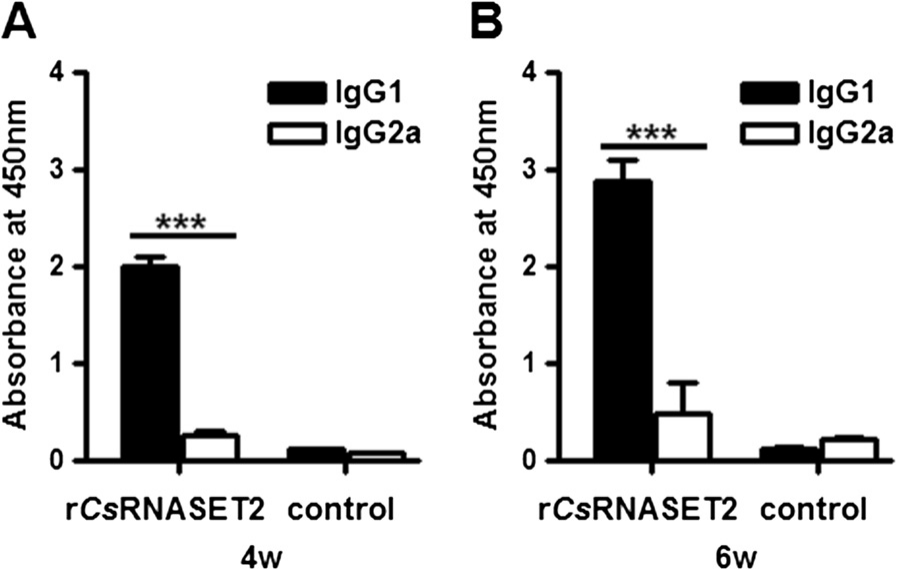
**IgG subclass responses triggered by r*Cs*RNASET2.** BALB/c mice (n = 6) were immunized s.c. with r*Cs*RNASET2. After 4 **(A)** and 6 **(B)** weeks, sera levels of IgG1 and IgG2a specific for r*Cs*RNASET2 were evaluated by ELISA. IgG1 levels were significantly higher than IgG2a at both 4th and 6th week. Error bars represent mean levels ± SD. Statistical significance was analyzed by the Mann–Whitney test (***p < 0.0005).

For mice, the IgG1 response generally represents Th2-activity, while IgG2a represents Th1-activity [29]. We evaluated the levels of IgG1 and IgG2a in sera obtained from r*Cs*RNASET2 immunized mice. The data shown in Figure 5 shows that administration of r*Cs*RNASET2 triggered a markedly higher IgG1 expression than IgG2a compared with control mice at week 4 (1.99 ± 0.12 versus 0.26 ± 0.04, *p* < 0.0001) and week 6 (2.88 ± 0.22 versus 0.49 ± 0.31, *p* = 0.0002).

## Discussion

It is well demonstrated that DCs are dominant players in the initiation and sustenance of immune responses [19-22]. In general, DCs undergo activation, termed maturation, upon recognition of invading pathogens, such as viruses, bacteria, and fungi. In the process of maturation, DCs up-regulate the production of surface markers and polarizing cytokines [20,21]. However, recent studies have indicated that DCs exposed to antigens derived from parasitic helminths fail to induce the classical DC activation [30-32]. The maturation status of DCs is crucial for the initiation of primary immune responses. It has been documented that *Sm*omega-1, a RNase T2 family glycoprotein present in ESP, is a major molecule to dramatically modulate the maturation of DCs in *S. mansoni* infections [14-17].

Since *Sm*omega-1 has been reported to play a potent role in the process of DCs modulation, we wondered whether there was a counterpart present in *Cs*ESPs with similar activity. In this study, we firstly identified a gene encoding *Cs*RNASET2, which also appeared to be a glycoprotein of RNase T2 family. All RNase T2 enzymes have two blocks of conserved amino acids (termed CAS I and CAS II) [8,9]. These regions have typical catalytic residue of one to three histidine residues [8,33]. Sequence analysis showed that *Cs*RNASET2 indeed harbored the two conserved CAS I and CAS II regions with two critical histidine residues (Figure 1). It suggested that *Cs*RNASET2 was the counterpart of *Sm*omega-1. We expressed r*Cs*RNASET2 in *P. pastoris* with RNase activity. Deglycosylation experiment displayed ~4 kDa size reduction of the r*Cs*RNASET2 (Figure 2A), which indicated the r*Cs*RNASET2 was indeed glycosylated. r*Cs*RNASET2 could react with *C. sinensis*-infected mouse sera and ESPs immunized mouse sera, meanwhile, ESPs could be probed with mouse anti-r*Cs*RNASET2 sera at a single band (Figure 2B). These results revealed that *Cs*RNASET2 was indeed a component of *Cs*ESPs, which are in accordance with the fact that the RNase T2 family members are typically secreted [9,34]. The difference of 8 kDa in molecular weight between r*Cs*RNASET2 and the native protein in ESP might be due to his/myc-tag, a minor imprecision on SDS-PAGE assays and glycosylation (~4 kDa reduction). Meanwhile, we performed western blot analysis, in which ESP reacted with anti-r*Cs*RNASET2 and anti-ESP mouse sera. Gradation analysis revealed that *Cs*RNASET2 took up about 2% of ESP (Additional file 1: Figure S3).

Furthermore, ELISA assays revealed that the LPS-induced up-regulation of IL-12p70 on BMDCs decreased in a dose-dependent manner in the presence of various concentrations of r*Cs*RNASET2 or *Cs*ESP (Figure 3A). In contrast, *Cs*ESPs (20–80 μg/ml) enhanced the expression of IL-10 in a dose-dependent manner, and r*Cs*RNASET2 could promote IL-10 synthesis only at a concentration of 0.5 μg/ml. Higher concentrations of r*Cs*RNASET2 or ESP failed to promote IL-10 production (Figure 3B). We then performed BMDCs survival assay with Cell Counting kit-8 (CCK8) under different concentrations of r*Cs*RNASET2 or ESP, and observed that higher concentrations of r*Cs*RNASET2 or ESP could inhibit cell viability (Additional file 1: Figure S4). Therefore, we finally chose 0.5 μg/ml of r*Cs*RNASET2 and 40 μg/ml of ESP as moderate working concentrations. *Cs*FABP, a Th1-polarizing Ag, was utilized to be a control protein. There was no significant difference of cytokine production levels when r*Cs*FABP was added. Subsequently, we carried out flow cytometry analysis and noted that levels of surface markers including CD11C, CD40, CD80 and CD86 on r*Cs*RNASET2- or *Cs*ESP-treated BMDCs significantly decreased compared with LPS controls, whereas r*Cs*FABP displayed little effect on these surface makers expression (Figure 4). These results demonstrated that r*Cs*RNASET2 could modulate the cytokine production and maturation of DCs. Interleukin-12 (IL-12) plays the key role in the generation of Th1 cells. IL-12p70 is a heterodimeric cytokine composed of two chains (p35 and p40), which is able to stimulate NK cells and T cells to produce IFN-γ to resist against pathogens [35]. It has been well established that IL-10 is a cytokine secreted by CD4^+^ T cells belonging to the Th2 subset, which has been determined to suppress the production of IFN-γ. The induction of IL-10 may be an important strategy by which parasites evade IFN-γ-dependent, cell-mediated immune destruction [36]. Our experiments showed diminished expression of IL-12p70 and increased expression of IL-10 in r*Cs*RNASET2-cocultured DCs. Therefore, it is conceivable that r*Cs*RNASET2 co-cultured DCs may suppress Th1 polarization, which is eventually beneficial for *C. sinensis* escaping from the host immunity.

IgG2a and IgGl immunoglobulin isotypes are generally regarded as markers for Th1 and Th2 responses respectively [29,37]. Our studies demonstrated that the up-regulation of IgG1 was greater than that of IgG2a (Figure 5), confirming that Th2 responses might be dominant in r*Cs*RNASET2 immunized mice. These results were in agreement with the data obtained from r*Cs*RNASET2 co-incubated BMDCs, as reduced expression of IL-12p70 and increased production of IL-10 could inhibit Th1 polarization. However, it is difficult to determine the dominance of Th1 or Th2 responses on the basis of IgG isotypes alone, because the initiation of B cell isotype switching is not only confined to the mutual effects of Th1 and Th2 cytokines. Further studies are required to determine whether *Cs*RNASET2 is able to trigger Th2 or Treg responses through DCs modulation, whether this function is associated with its RNase activity, and which pathways are involved in the process.

## Conclusion

Collectively, we cloned and identified *Cs*RNASET2 as a molecule present in *C. sinensis* ESPs. We confirmed that r*Cs*RNASET2 could regulate the host immune response via modulating DCs maturation, as well as production of IL-12p70 and IL-10. We also observed that administration of r*Cs*RNASET2 to mice could alter the IgG1/IgG2a ratio. It will be of significant interest to investigate whether *Cs*RNASET2 is able to initiate Th2 or Treg responses through modulating DCs, which will pave the way towards unraveling the regulatory mechanisms of the immune responses in *C. sinensis* infection and may provide potential therapeutic tools.

## Competing interests

The authors declare that they have no competing interests.

## Authors’ contributions

YQX, XNZ, YH and XBY conceived and designed the experiments; YQX, WJC, MB, XYW, JFS, HCS, FFJ, CL, XRL performed the experiments; YQX, WJC and MB analyzed the data; YQX, WJC, XYW and YH wrote the manuscript; All authors read and approved the final manuscript.

## Supplementary Material

Additional file 1: Figure S1 Expression and purification of r*Cs*RNASET2. **Figure S2.** Determination of ribonuclease activity of r*Cs*RNASET2. **Figure S3.** Relative quantification of *Cs*RNASET2 in *Cs*ESP. **Figure S4.** Cytotoxity assessment of r*Cs*RNASET2. Click here for file

## References

[B1] Lin RQ Tang JD Zhou DH Song HQ Huang SY Chen JX Chen MX Zhang H Zhu XQ Zhou XN Prevalence of *Clonorchis sinensis* infection in dogs and cats in subtropical southern China Parasit Vectors 2011 6 180 21929783 10.1186/1756-3305-4-180 PMC3183008

[B2] Chen J Xu MJ Zhou DH Song HQ Wang CR Zhu XQ Canine and feline parasitic zoonoses in China Parasit Vectors 2012 6 152 22839365 10.1186/1756-3305-5-152 PMC3431282

[B3] Kim HG Han J Kim MH Cho KH Shin IH Kim GH Kim JS Kim JB Kim TN Kim TH Kim TH Kim JW Ryu JK Moon YS Moon JH Park SJ Park CG Bang SJ Yang CH Yoo KS Yoo BM Lee KT Lee DK Lee BS Lee SS Lee SO Lee WJ Cho CM Joo YE Cheon GJ Prevalence of clonorchiasis in patients with gastrointestinal disease: a Korean nationwide multicenter survey World J Gastroenterol 2009 6 86 94 19115472 10.3748/wjg.15.86 PMC2653299

[B4] Keiser J Utzinger J Food-borne trematodiases Clin Microbiol Rev 2009 6 466 483 19597009 10.1128/CMR.00012-09 PMC2708390

[B5] Lei HL Tian YL Chen WJ Wang XY Li XR Mao Q Sun JF Li R Xu YQ Liang C Huang Y Yu XB The biochemical and immunological characterization of two serpins from *Clonorchis sinensis* Mol Biol Rep 2013 6 3977 3985 23275238 10.1007/s11033-012-2475-1

[B6] Qian MB Chen YD Fang YY Xu LQ Zhu TJ Tan T Zhou CH Wang GF Jia TW Yang GJ Zhou XN Disability weight of *Clonorchis sinensis* infection: captured from community study and model simulation PLoS Negl Trop Dis 2011 6 e1377 22180791 10.1371/journal.pntd.0001377 PMC3236727

[B7] Fried B Reddy A Mayer D Helminths in human carcinogenesis Cancer Lett 2011 6 239 249 20667649 10.1016/j.canlet.2010.07.008

[B8] Luhtala N Parker R T2 Family ribonucleases: ancient enzymes with diverse roles Trends Biochem Sci 2010 6 253 259 20189811 10.1016/j.tibs.2010.02.002 PMC2888479

[B9] Deshpande RA Shankar V Ribonucleases from T2 family Crit Rev Microbiol 2002 6 79 122 12109772 10.1080/1040-840291046704

[B10] Schwartz B Shoseyov O Melnikova VO McCarty M Leslie M Roiz L Smirnoff P Hu GF Lev D Bar-Eli M ACTIBIND, a T2 RNase, competes with angiogenin and inhibits human melanoma growth, angiogenesis and metastasis Cancer Res 2007 6 5258 5266 17545605 10.1158/0008-5472.CAN-07-0129

[B11] Dunne DW Jones FM Doenhoff MJ The purification, characterization, serological activity and hepatotoxic properties of two cationic glycoproteins (alpha 1 and omega 1) from *Schistosoma mansoni* eggs Parasitology 1991 6 225 236 1745548 10.1017/s0031182000059503

[B12] Fitzsimmons CM Schramm G Jones FM Chalmers IW Hoffmann KF Grevelding CG Wuhrer M Hokke CH Haas H Doenhoff MJ Dunne DW Molecular characterization of omega-1: a hepatotoxic ribonuclease from *Schistosoma mansoni* eggs Mol Biochem Parasitol 2005 6 123 127 16143411 10.1016/j.molbiopara.2005.08.003

[B13] Meevissen MH Wuhrer M Doenhoff MJ Schramm G Haas H Deelder AM Hokke CH Structural characterization of glycans on omega-1, a major *Schistosoma mansoni* egg glycoprotein that drives Th2 responses J Proteome Res 2010 6 2630 2642 20178377 10.1021/pr100081c

[B14] Everts B Hussaarts L Driessen NN Meevissen MH Schramm G van der Ham AJ van der Hoeven B Scholzen T Burgdorf S Mohrs M Pearce EJ Hokke CH Haas H Smits HH Yazdanbakhsh M Schistosome-derived omega-1 drives Th2 polarization by suppressing protein synthesis following internalization by the mannose receptor J Exp Med 2012 6 1753 1767 22966004 10.1084/jem.20111381 PMC3457738

[B15] Everts B Perona-Wright G Smits HH Hokke CH van der Ham AJ Fitzsimmons CM Doenhoff MJ van der Bosch J Mohrs K Haas H Mohrs M Yazdanbakhsh M Schramm G Omega-1, a glycoprotein secreted by *Schistosoma mansoni* eggs, drives Th2 responses J Exp Med 2009 6 1673 1680 19635864 10.1084/jem.20082460 PMC2722183

[B16] Steinfelder S Andersen JF Cannons JL Feng CG Joshi M Dwyer D Caspar P Schwartzberg PL Sher A Jankovic D The major component in schistosome eggs responsible for conditioning dendritic cells for Th2 polarization is a T2 ribonuclease (omega-1) J Exp Med 2009 6 1681 1690 19635859 10.1084/jem.20082462 PMC2722182

[B17] Zaccone P Burton OT Gibbs SE Miller N Jones FM Schramm G Haas H Doenhoff MJ Dunne DW Cooke A The *S. mansoni* glycoproteinω-1 induces Foxp3 expression in NOD mouse CD4^+^T cells Eur J Immunol 2011 6 2709 2718 21710488 10.1002/eji.201141429

[B18] Banchereau J Steinman RM Dendritic cells and the control of immunity Nature 1998 6 245 252 9521319 10.1038/32588

[B19] Steinman RW Hawiger D Nussenzweig MC Tolerogenic dendritic cells Annu Rev Immunol 2003 6 685 711 12615891 10.1146/annurev.immunol.21.120601.141040

[B20] Banchereau J Briere F Caux C Davoust J Lebecque S Liu YJ Pulendran B Palucka K Immunobiology of dendritic cells Annu Rev Immunol 2000 6 767 811 10837075 10.1146/annurev.immunol.18.1.767

[B21] Lipscomb MF Masten BJ Dendritic cells: immune regulators in health and disease Physiol Rev 2002 6 97 130 11773610 10.1152/physrev.00023.2001

[B22] Morelli AE Thomson AW Tolerogenic dendritic cells and the quest for transplant tolerance Nat Rev Immunol 2007 6 610 621 17627284 10.1038/nri2132

[B23] Chen WJ Wang XY Li XR Lv XL Zhou CH Deng CH Lei HL Men JT Fan YX Lian C Yu XB Molecular characterization of cathepsin B from *Clonorchis sinensis* excretory/secretory products and assessment of its potential for serodiagnosis of clonorchiasis Parasit Vectors 2011 6 149 21794140 10.1186/1756-3305-4-149 PMC3163202

[B24] Cervi L MacDonald AS Kane C Dzierszinski F Pearce EJ Cutting edge: dendritic cells copulsed with microbial and helminth antigens undergo modified maturation, segregate the antigens to distinct intracellular compartments, and concurrently induce microbe-specific Th1 and helminth-specific Th2 responses J Immunol 2004 6 2016 2020 14764665 10.4049/jimmunol.172.4.2016

[B25] Lee JS Kim IS Sohn WM Lee J Yong TS A DNA Vaccine Encoding a Fatty Acid-Binding Protein of *Clonorchis sinensis* Induces Protective Immune Response in Sprague–Dawley Rats Scand J Immunol 2006 6 169 176 16499569 10.1111/j.1365-3083.2006.01721.x

[B26] Trinchieri G Pflanz S Kastelein RA The IL-12 family of heterodimeric cytokines: new players in the regulation of T cell responses Immunity 2003 6 641 644 14614851 10.1016/s1074-7613(03)00296-6

[B27] Moore KW de Waal MR Coffman RL O’Garra A Interleukin-10 and the interleukin-10 receptor Annu Rev Immunol 2001 6 683 765 11244051 10.1146/annurev.immunol.19.1.683

[B28] Everts B Smits HH Hokke CH Yazdanbakhsh M Helminths and dendritic cells: sensing and regulating via pattern recognition receptors, Th2 and Treg responses Eur J Immunol 2010 6 1525 1537 20405478 10.1002/eji.200940109

[B29] Mountford AP Fisher A Wilson RA The profile of lgGl and IgG2a antibody responses in mice exposed to *Schistosoma mansoni* Parasite Immunol 1994 6 521 527 7870462 10.1111/j.1365-3024.1994.tb00306.x

[B30] Jeong YI Kim SH Ju JW Cho SH Lee WJ Park JW Park YM Lee SE *Clonorchis sinensis*-derived total protein attenuates airway inflammation in murine asthma model by inducing regulatory T cells and modulating dendritic cell functions Biochem Biophys Res Commun 2011 6 793 800 21440530 10.1016/j.bbrc.2011.03.102

[B31] MacDonald AS Straw AD Bauman B Pearce EJ CD8^-^ dendritic cell activation status plays an integral role in influencing Th2 response development J Immunol 2001 6 1982 1988 11489979 10.4049/jimmunol.167.4.1982

[B32] MacDonald AS Straw AD Dalton NM Pearce EJ Cutting edge: Th2 response induction by dendritic cells: a role for CD40 J Immunol 2002 6 537 540 11777943 10.4049/jimmunol.168.2.537

[B33] Kawata Y Sakiyama F Hayashi F Kyogoku Y Identification of two essential histidine residues of ribonuclease T2 from Aspergillus oryzae Eur J Biochem 1990 6 255 262 2298207 10.1111/j.1432-1033.1990.tb15303.x

[B34] Campomenosi P Salis S Lindqvist C Mariani D Nordström T Acquati F Taramelli R Characterization of RNASET2, the first human member of the Rh/T2/S family of glycoproteins Arch Biochem Biophys 2006 6 17 26 16620762 10.1016/j.abb.2006.02.022

[B35] Hunter CA New IL-12-family members: IL-23 and IL-27, cytokines with divergent functions Nat Rev Immunol 2005 6 521 531 15999093 10.1038/nri1648

[B36] Ouyang W Rutz S Crellin NK Valdez PA Hymowitz SG Regulation and functions of the IL-10 family of cytokines in inflammation and disease Annu Rev Immunol 2011 6 71 109 21166540 10.1146/annurev-immunol-031210-101312

[B37] Maassen CBM Boersma WJA van Holten-Neelen C Claassen E Laman JD Growth phase of orally administered *Lactobacillus* strains differentially affects IgG1/IgG2a ratio for soluble antigens: implications for vaccine development Vaccine 2003 6 2751 2757 12798614 10.1016/s0264-410x(03)00220-2

