# Supplementary material for: Molecular characterization and immune modulation properties of Clonorchis sinensis-derived RNASET2
Source: Parasit Vectors. 2013 Dec 23;6:360. doi: 10.1186/1756-3305-6-360 (PMC3878043; doi:10.1186/1756-3305-6-360)
Supplement: Additional file 1: Figure S1 — Expression and purification of rCsRNASET2. Figure S2. Determination of ribonuclease activity of rCsRNASET2. Figure S3. Relative quantification of CsRNASET2 in CsESP. Figure S4. Cytotoxity assessment of rCsRNASET2. [file 1756-3305-6-360-S1.doc]

**
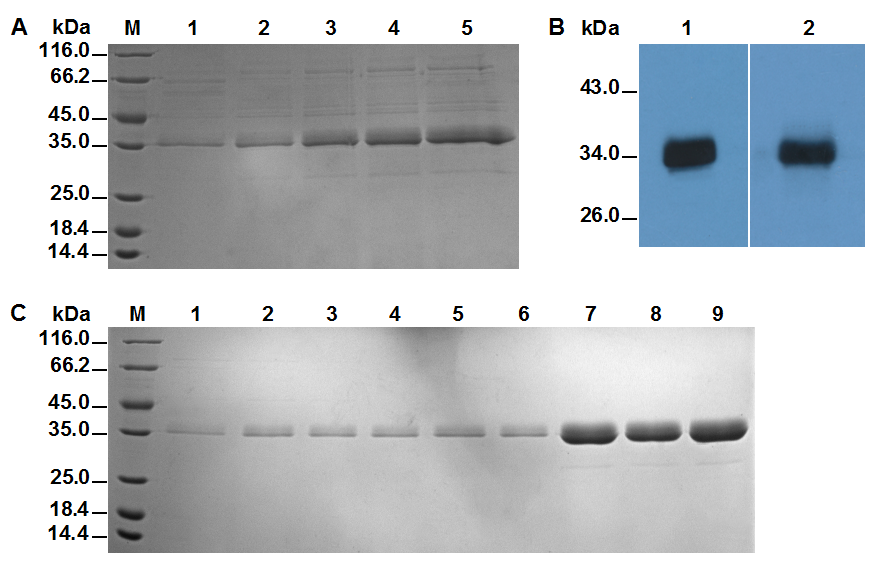
**

**Figure. S1 Expression and purification of r*Cs*RNASET2.** Gene sequence encoding full-length *Cs*RNASET2 was amplified by PCR, and then cloned into the vector pPICZαB (Invitrogen, USA). The recombinant plasmid was propagated in DH5α (*E. coli*). Subsequently, the purified plasmids were transformed into *P. pastoris* strain X-33. Finally, a single colony was cultured in BMMY medium with 0.5% methanol to express r*Cs*RNASET2. (A) Expression culture collected at 24 (lane 1), 48 (lane 2), 72 (lane 3), 96 (lane 4) and 120 h (lane 5) were detected by 12 % SDS-PAGE. (B) Western blot analysis. r*Cs*RNASET2 samples were immobilized onto the membrane and then confirmed by anti-myc (lane 1), anti-his (lane 2) monoclonal antibodies; (C) Purified r*Cs*RNASET2 samples were analyzed by 12 % SDS-PAGE. lane M : protein molecular weight markers , lane 1-9: the purified r*Cs*RNASET2eluted using a concentration range of imidazole containing buffer. One of three independent experiments is shown.


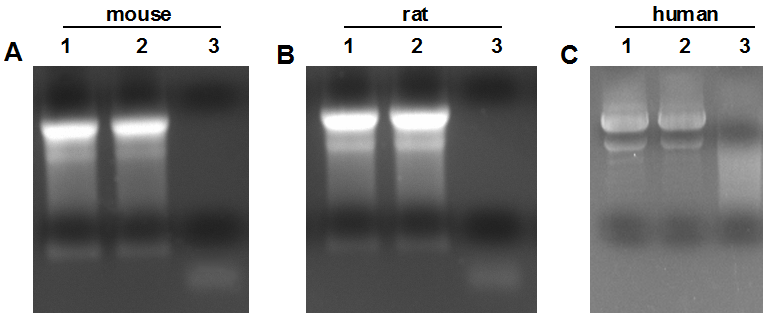


**Figure. S2 Determination of ribonuclease activity of r*Cs*RNASET2.** Total RNA from mouse (A), rat (B) splenocytes and human (C) PBMCs respectively were incubated with 1 μg/ml BSA (lane 1), DEPC pre-treated r*Cs*RNASET2 (lane 2) or r*Cs*RNASET2 (lane 3) at 37°C for 1h and running on a 2% agarose gel for degradation. BSA was used as a negative control. One of three independent experiments is shown.


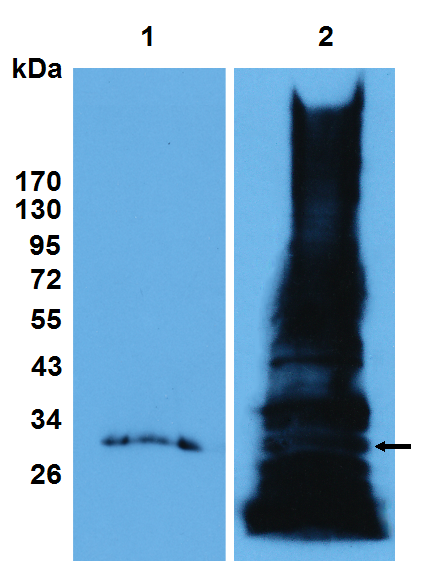


**Figure S3 R**[**elative**](app:ds:relative) [**quantification**](app:ds:quantification) **of *Cs*RNASET2 in *Cs*ESP.** Western blot analysis was performed, in which ESP reacted with anti-r*Cs*RNASET2 (lane 1) or anti-ESP (lane 2) mouse sera. Gradation analysis revealed that *Cs*RNASET2 took up about 2% of ESP.


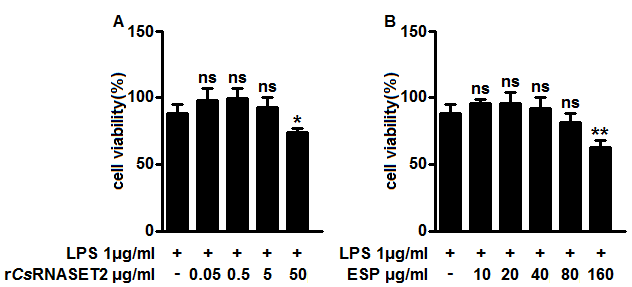


**Figure. S4 Cytotoxity assessment of r*Cs*RNASET2.** BMDCs were cultured with LPS in the presence or absence of different concentrations of r*Cs*RNASET2 (A) or ESPs (B) for 48 h, and then BMDCs survival was detected using Cell Counting Kit-8. Data are expressed as mean ± SD. Statistical significance was analyzed by the Mann–Whitney test (**p* < 0.05, ns: not significant).
